# Recent Advances in Dopamine-Based Membrane Surface Modification and Its Membrane Distillation Applications

**DOI:** 10.3390/membranes14040081

**Published:** 2024-03-28

**Authors:** Haodong Jia, Jing Ren, Yue Kong, Zhongjia Ji, Shujuan Guo, Jianfeng Li

**Affiliations:** Shanxi Laboratory for Yellow River, Institute of Resources and Environmental Engineering, Shanxi University, Taiyuan 030006, China; 202214001004@email.sxu.edu.cn (H.J.); 202124002019@email.sxu.edu.cn (Y.K.); 202124001009@email.sxu.edu.cn (Z.J.); 202014001002@email.sxu.edu.cn (S.G.)

**Keywords:** membrane modification, dopamine, membrane distillation, mechanisms

## Abstract

Surface modification of membranes is essential for improving flux and resistance to contamination for membranes. This is of great significance for membrane distillation, which relies on the vapor pressure difference across the membrane as the driving force. In recent years, biomimetic mussel-inspired substances have become the research hotspots. Among them, dopamine serves as surface modifiers that would achieve highly desirable and effective membrane applications owing to their unique physicochemical properties, such as universal adhesion, enhanced hydrophilicity, tunable reducibility, and excellent thermal conductivity. The incorporation of a hydrophilic layer, along with the utilization of photothermal properties and post-functionalization capabilities in modified membranes, effectively addresses challenges such as low flux, contamination susceptibility, and temperature polarization during membrane distillation. However, to the best of our knowledge, there is still a lack of comprehensive and in-depth discussions. Therefore, this paper systematically compiles the modification method of dopamine on the membrane surface and summarizes its application and mechanism in membrane distillation for the first time. It is believed that this paper would provide a reference for dopamine-assisted membrane separation during production, and further promote its practical application.

## 1. Introduction

Membrane distillation (MD) is a membrane separation process in which the steam pressure difference between the two sides of the hydrophobic microporous membrane is the driving force of mass transfer. It is a promising process in the water–energy relationship. It can obtain high quality water at relatively low temperatures and pressure. Theoretically, only water vapor can pass through the membrane pore, and it can achieve 100% interception of ions, macromolecules, colloids, cells and other non–volatile substances [1,2,3]. Moreover, it can collect renewable energy, including solar energy, geothermal energy and low–grade waste heat, as a heat source to provide heat for the feed side, which means that the process has higher energy efficiency and lower operating costs [4,5,6]. Due to these advantages of membrane distillation, the process has great application potential in the fields of seawater desalination, wastewater treatment, ultrapure water production, the food industry and the medical industry [7,8].

However, membrane distillation has not been widely applied at present, and one of the main reasons is the wetting and pollution problems. These problems in the operation of membrane distillation will reduce the passage of steam through, affect the membrane mass transfer, deteriorate the effluent quality and reduce the separation efficiency [9,10]. Currently, researchers have realized the surface modification of MD membranes with radiation graft polymerization, plasma polymerization, grafting ceramic membranes, hydrophobic or hydrophilic surface coating, casting hydrophobic polymers over flat sheet or porous fibres as supports, co–extrusion spinning and the use of surface–modifying macromolecules [11,12]. Among them, hydrophobic or hydrophilic coating is the most common technique used for MD modified membranes, which has simpler and less costly steps [13]. Zhang et al. [14] configured hydrophobic nano–SiO_2_ with polydimethylsiloxane (PDMS) as a coating solution and sprayed it onto a hydrophobic polyvinylidene fluoride (PVDF) flat film to form a superhydrophobic coating. The membrane has excellent resistance to contamination during MD operation. Gupta et al. [15] immobilized carbon nanotubes (CNTs), graphene oxide (GO), reduced graphene oxide (rGO) and an rGO−CNT hybrid on polytetrafluoroethylene membranes and used membrane distillation for the separation and recovery of tetrahydrofuran (THF) in water, and the performance of all the modified membranes was significantly improved. However, many of the modified materials and adhesives used in the modification process are expensive and not environmentally friendly, which is not conducive to the promotion and utilization of modified films.

Dopamine (DA) is a representative mussel bionic material and good adherent substance. In 2007, Lee et al. [16] discovered that dopamine can undergo oxidative self–polymerization in a weak alkaline environment and formed a polydopamine (PDA) coating with strong adhesion on different substrate surfaces. Dopamine, a biomimetic modifier with universal adhesion, hydrophilic properties, easy processing and nonmspecific adsorption, is widely used in the modification of various membrane surfaces. Due to their easy preparation, excellent hydrophilic property, surface functionalization and photothermal properties, dopamine–modified membranes are also of great interest to researchers in the field of membrane distillation [17,18]. 

Therefore, we summarised the various modification methods of dopamine deposition on membrane surfaces, and provided the first overview of the application and mechanism of action of dopamine in membrane distillation. Firstly, this paper introduces the current various methods of dopamine deposition modification on membrane surfaces and discusses the advantages and disadvantages of these methods. Then, it introduces the current status of using dopamine to enhance the performance of membrane distillation at home and abroad and summarises the mechanism of performance enhancement. Finally, the prospect of dopamine in the process of membrane distillation application is elaborated to provide a reference for the research and application of dopamine in membrane distillation.

## 2. Dopamine Membrane Surface Modification

Membrane technology applications have always been a research hotspot, and although much progress has been made, there are still some problems with current membrane applications. First, the flux of membrane material is too low to meet a wide range of industrial production applications [13,19]; second, the anti–fouling performance and durability of the membrane cannot support its long–term operation. The high cost and poor reusability limit its application in actual production and life [20,21,22]. Nowadays, with the gradual improvement in membrane process theory, researchers seek new membrane materials and methods to modify membrane surfaces to improve membrane performance and maximize the efficiency of industrial applications [23]. 

Dopamine has a universal adhesion property and is capable of forming polydopamine coatings on a variety of substrates including precious metals, oxides, polymers, semiconductors and ceramics [16]. This property has attracted the interest of a large number of researchers. In the environmental field, combining PDA coatings with high specific surface area materials can construct high–performance adsorbent materials for the treatment of heavy metal and dye wastewater; combining with polymer separation membrane materials, it can be widely used in environmental remediation and has great potential in separation fields such as aqueous nanofiltration membranes, osmotic vaporization membranes and gas separation membranes [24,25]. Currently, researchers have proposed new membrane surface modification technologies, such as rapid polymerization deposition modification of induced dopamine, co–deposition modification of dopamine and secondary modification of dopamine film surfaces. These new membrane surface modification technologies alleviate the problems of traditional PDA membranes such as long deposition time, poor coating uniformity and easy peeling off in strong acid and alkali solutions, and improve the application range of PDA membranes [26]. In this section, the surface modification method of the dopamine membrane will be analysed to provide some references and ideas.

### 2.1. Traditional Deposition Modification of Dopamine

The conventional method for surface deposition modification of dopamine membranes is to immerse the substrate in an alkaline dopamine solution, where the dopamine polymerizes on the surface of the solid material in the presence of oxygen in the air to produce a thin layer of polydopamine [27]. After some time, the substrate is taken out to dry and a layer of PDA–modified material can be attached to the surface of the substrate. Xi et al. [28] immersed polyethylene (PE), polyvinylidene fluoride (PVDF) and polytetrafluoroethylene (PTFE) hydrophobic porous polymer membranes in a dopamine solution for surface hydrophilic modification and investigated in detail the effects of different modification conditions such as deposition time, solution concentration and pH on membrane structure and properties, and finally provided a general method for surface modification of various polymer membranes. The conventional dopamine deposition modification method uses oxygen in the air to create an oxidizing environment, and the modification process has the advantages of mild reaction conditions, simple operation and no selectivity on the chemical properties and shape of the membrane surface [29,30]. However, this conventional deposition technique has some drawbacks, such as long deposition reaction time, poor coating uniformity and poor stability in acids, bases and polar solvents [31,32,33]. These problems greatly limit the practical application of polydopamine coatings on a large scale.

### 2.2. Induce Rapid Deposition Modification of Dopamine

**Figure 1:**
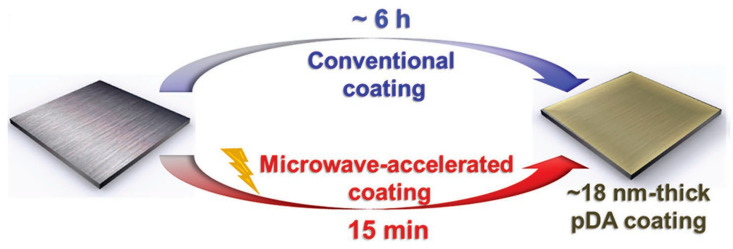
Schematic diagram of PDA microwave–induced deposition compared with conventional deposition. Reprinted with permission from ref. [41]. copyright John Wiley and Sons, 2016.

To address the problems in the traditional dopamine deposition process, researchers have used various methods to induce rapid polymerization of dopamine deposition. Zhang et al. [34] used CuSO_4_/H_2_O_2_ as an inducer to change the oxidation environment of conventional dopamine solution deposition, which greatly accelerated the polymerization of dopamine and the deposition of polydopamine coating, making the deposition time shortened to 40 min and achieving rapid deposition of PDA on the surface of a polypropylene (PP) microporous membrane. Moreover, the membranes prepared with rapid deposition exhibited high uniformity and stability, and the addition of Cu^2+^ conferred excellent bactericidal properties to the coatings. Correspondingly, ammonium persulfate [35], potassium permanganate [36], sodium periodate [37] and persulfate [38] have also been introduced to alter the oxidative environment of dopamine deposition and induce rapid dopamine polymerization. However, during the process of dopamine deposition, these chemical oxidants inevitably interact with catechol and amino groups of dopamine, resulting in the contamination of the PDA membrane. So, some scientists have found physical ways to speed up dopamine deposition without contaminating the PDA membrane. Du et al. [39] used microwave radiation to induce rapid polymerization of dopamine, and under the action of microwaves as in Figure 1, a PDA coating of about 18 nm could be produced on the material surface within 15 min, which is a significant improvement compared to the conventional deposition rate (about a 50 nm PDA deposition thickness obtained in 18 h). Microwave–induced free radicals in the aqueous solution are a key factor that greatly improves the polymerization rate of PDA. In addition, rapid polymerization of dopamine without the addition of oxidants can also be achieved with methods such as an electric field [40] and UV irradiation [41]. These methods for inducing rapid dopamine deposition not only greatly reduce the dopamine deposition time but also improve the homogeneity and stability of polydopamine coatings.

### 2.3. Dopamine Co–Deposition Modification

The dopamine co–deposition process is a process in which another component (e.g., polymers [42], biomolecules [43], nanomaterials [44] and inorganic precursors [45]) is added to the dopamine deposition process to enhance the performance of the polydopamine modified film using covalent or non–covalent interactions with dopamine to achieve co–deposition on the surface of the material; the process can be classified as non–covalent co–deposition [46], covalent co–deposition [26] and precursor–involved co–deposition [47] with the different components of the co–deposition. Zhou et al. [48] prepared microporous polypropylene anti–fouling membranes using co–depositing poly(sulfobetaine methacrylate) (PSBMA) with dopamine on the surface of polypropylene membranes based on the self–polymerization and high adhesion of dopamine, taking advantage of the excellent anti–fouling properties of PSBMA. Dynamic protein filtration experiments show that the prepared membrane has good anti–fouling performance, and the PSBMA/PDA co–deposition coating also shows good stability in longterm operation. This dopamine co–deposition modification method provides a simple and effective way to construct anti–fouling surfaces for membranes. Yang et al. [26] co–deposited polydopamine and polyethyleneimine (PEI) on polypropylene microfiltration membranes and the prepared membranes showed significantly improved surface hydrophilicity and excellent water permeation fluxes. The covalent crosslinking between PDA and PEI endowed the co–modified membrane with better stability in a strong alkali or acid environment. In addition, the addition of PEI during the deposition of dopamine made the deposition time of the film much shorter, and the PDA/PEI deposition solution could be reused many times with almost no effect on the surface hydrophilicity of the prepared membrane, avoiding the waste of the drug during the deposition modification. Although the complexity of the dopamine co–deposition process makes the mechanism unclear, this technique has great potential in the field of material surface engineering and is expected to gradually become one of the conventional means of material surface modification in future research.

### 2.4. Secondary Modification of Dopamine Membrane Surface

**Figure 2:**
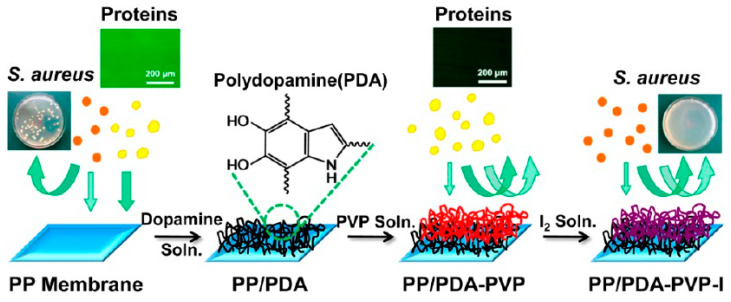
Schematic diagram of the PP–based PDA modification and the subsequent modification scheme with PVP and iodine. Reprinted with permission from ref. [58]. Copyright American Chemica Society, 2013.

Polydopamine deposits have some specific functional groups such as indole, catechol, o–dibenzoquinones and amino and carboxyl groups [49], indicating that they are rich in modification sites and possess good post–functionalization capabilities to introduce synthetic polymers [50], inorganic nanoparticles [51], proteins [52], polysaccharides [53], growth factors [54] and hydroxyapatite [55] onto the surface of the material through non–covalent interactions, covalent coupling and surface initiated reactive radical polymerization [56,57]. The pervasive bonding and excellent post–functionalization ability of PDA point to the idea of improving the performance of some chemically inert materials. Jiang et al. [58] performed surface modification of hydrophobic PP membranes with PDA, while the surface of PDA was further modified using multiple hydrogen bonding between poly–N–vinylpyrrolidone (PVP) and PDA, and finally the antimicrobial iodine was introduced to the membrane through complexation with the PVP layer (Figure 2). A series of surface modifications greatly improved the anti–fouling and antibacterial properties of PP membranes. Hou et al. [59] used PTFE as a reinforcing material to enhance the mechanical properties of membranes in proton exchange membrane fuel cells, and used the bonding properties of PDA and abundant functional groups to load resveratrol on the membrane surface. Resveratrol is a natural phenolic antioxidant that acts as a free radical scavenger (FRS) in the membrane to prevent the membrane from being attacked by free radicals such as hydroxyl (·OH) and hydrogen (·H) generated on the cathode side of the cell, making the service life degraded. Compared with the original membrane, the life of the composite proton exchange membrane was extended by eight times, and the battery performance was lightly damaged. 

## 3. Application of Dopamine–Modified Membrane in Membrane Distillation

As a derivative of mussel adhesion protein, dopamine has many excellent physicochemical properties, which determine its application potential. The physical properties include hydrophilicity, adhesion, light absorption properties, etc., while the chemical properties are mainly reduction, nucleophilic reactions (Michael addition reaction and Schiff base reaction), etc. These properties play a key role in improving the performance of membrane distillation and expanding the application range of membrane distillation. Next, the application of dopamine in membrane distillation will be discussed in terms of its physicochemical properties.

### 3.1. The Adhesion Property

Dopamine has universal bonding properties and can be easily attached to hydrophobic membrane surfaces for hydrophilic modification to prepare hydrophilic and hydrophobic composite membranes to enhance membrane distillation performance. Researchers used two commonly used techniques, atomic force microscopy (AFM) [60] and a surface force apparatus (SFA) [61], to study the adhesion of PDA and investigated in detail the interaction of mussel mucin on different substrates and found that for different substrates, adhesion dominant forces differed [62]. The adhesion forces possessed by PDAs are generally considered to be non–covalent interaction forces, such as hydrophobic interactions, dipodal chelation (coordination), hydrogen bonding, cation–π interactions and π–π stacking, which may form covalent bonding forces with substances possessing amino groups. For different properties of the substrate, there will be one or more interaction forces as the adhesion dominant force. Dopamine interacts with metal and metal oxide surfaces such as Al_2_O_3_ and Fe_3_O_4_ predominantly using dibasic chelation, and with SiO_2_ surfaces predominantly using hydrogen bonding interactions [63,64,65]. For polymeric materials, the adhesion mechanism of PDA is more complex. For most polar polymers, electrostatic interactions and hydrogen bonding may be the dominant forces for adhesion, while for non–polar polymers such as polypropylene and polystyrene, π–π stacking or hydrophobic interactions are dominant [66]. 

**Figure 3:**
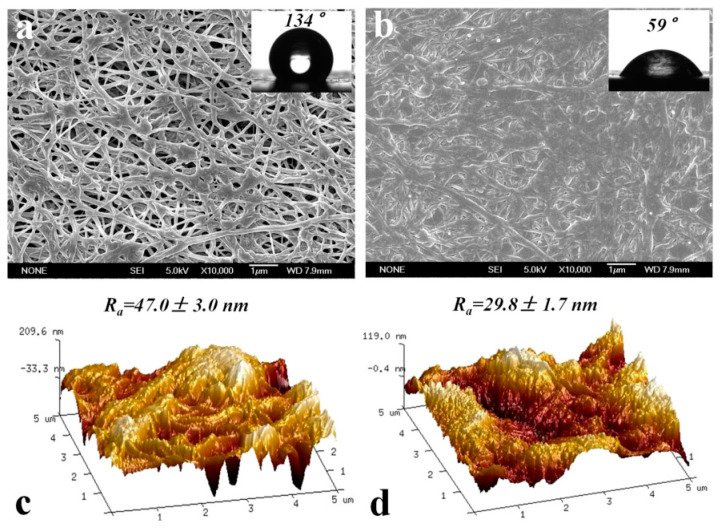
Membrane surface related characterization before and after PDA modification: (**a**) SEM and contact angle characterization before modification; (**b**) SEM and contact angle characterization after modification; (**c**) AFM characterization before modification; (**d**) AFM characterization after modification. Reprinted with permission from ref. [68]. Copyright Elsevier, 2018.

Sun et al. [67] used the bonding property of dopamine to attach GO to the surface of a polytetrafluoroethylene hydrophobic membrane, and provided a theoretical basis for the role of GO coating in enhancing the MD membrane flux by developing a mathematical model based on the capillary membrane hypothesis, and a series of molecular dynamics’ experimental results. Sun et al. [68] constructed an ultrathin, porous surface coating that is hydrophilic in air and oil–phobic in water by coating dopamine alone on a commercial PTFE membrane (Figure 3). When using vacuum membrane distillation (VMD) to treat a 3.5 wt% sodium chloride solution at 70 °C at a 92 kPa vacuum pressure, the modified membrane obtained three times more of a water flux than the original PTFE membrane, and was superior to the original membrane in the treatment of oily brine. In addition, the decrease in the surface roughness of the PDA–modified membrane reduces the deposition of NaCI crystallites on the membrane surface, which may also be the reason for the improved anti–pollution performance of the PDA–modified membrane in the salt solution.

### 3.2. The Reductive Property

Dopamine has a certain reducibility, and metal ion bonding is related to the functional groups of catechol, carboxyl, amino, imine and phenol in dopamine. Some noble metals such as Ag^+^, Au^+^, Pt^+^ and other metal ions can be reduced to metal nanoparticles under alkaline conditions, while the metal coordination ability of catechol in PDA can immobilize these reduced metal nanoparticles on the membrane surface [69]. The ability of dopamine to act as a reducing agent is largely related to the redox properties of its monomeric units. Studies have shown [70] that dopamine contains a certain amount of catechol groups, and when the catechol group is oxidized into the corresponding benzoquinone group, electrons can be released, thus triggering the reduction process of metal cations. In the formation of polydopamine, the oxidation of dopamine to dopamine–benzenediol releases two electrons, a process that reduces the metal salt to the corresponding metal. The reduction of metal ions to metal nanoparticles with dopamine requires neither additional reducing agents nor metal crystal seed particles. Moreover, the formed polydopamine prevents the agglomeration of metal nanoparticles due to hydroquinone and unoxidized catechol groups. In addition, the resulting polydopamine can also serve as an anchor site for the produced metal. The metal bonds formed using metal ions with N– and O– positions in polydopamine are the growth sites for metal nanoparticles, and with the continuous reduction of metal ions, the atoms keep growing to form metal nanoparticles [71,72]. 

**Figure 4:**
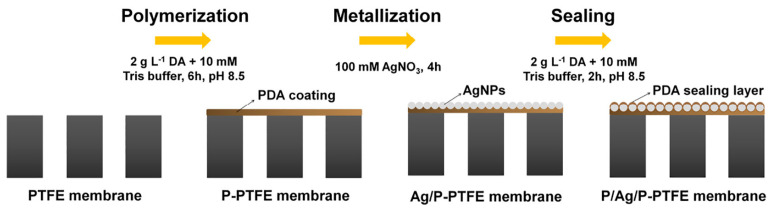
Schematic diagram of the in situ reductive immobilization of Ag^+^ on PTFE membrane surface with PDA. Reprinted with permission from ref. [74]. Copyright Elsevier, 2020.

Chew et al. [73] designed a new Janus MD membrane with a multi–level hierarchical structure on a commercial hydrophobic PVDF hollow fibre membrane modified using rapid polymerization of dopamine induced with oxidizing agents and reduction and in situ immobilization of Ag^+^ on the fibre membrane surface under shading conditions. The direct membrane distillation (DCMD) test obtained stable fluxes and excellent salt retention rates, with excellent contamination and wetting resistance, and exhibited antibacterial properties in acidic Bacillus solutions, which have a wide range of applications in membrane distillation. Zhang et al. [74] prepared a Janus membrane with an excellent structure by using the reducing property of dopamine to immobilize Ag^+^ in a silver nitrate solution in situ on the membrane surface under shading conditions and then again using dopamine for surface sealing to form an ultrathin PDA protective layer (Figure 4). The modified membrane achieved a 325.7% flux improvement when treating a 3.5 wt% NaCl solution (70 °C, −92 kPa) with the VMD device, from 19.8 ± 1.5 kg/(m^2^·h) to 84.3 ± 3.4 kg/(m^2^·h) with commercial PTFE membranes, while also showing long–term stability in treating oily brine. 

It is worth noting that the reductibility of dopamine is limited when complexing with Fe^3+^, Mn^2+^, Zn^2+^, Cu^2+^ and other polyvalent metal ions; that is, it cannot reduce the metal ions to metal nanoparticles. Under different pH conditions, the binding sites of dopamine to metal ions are different [75]. For example, the binding sites of Cu^2+^ in synthetic and naturally occurring true melanopsin were determined with electron paramagnetic resonance spectroscopy (ESR) using Cu^2+^ as a probe. The results showed the following [72,76]:

(1) At a pH of less than 5, Cu^2+^ forms complexes with carboxyl and bidentate nitrogen–carboxyl groups.
(2) At pH ≈ 7, the binding of Cu^2+^ to the phenol hydroxyl group was detected.
(3) With the further increase in pH, Cu^2+^ ions bind to three or four nitrogen ligands.

### 3.3. The Photothermal Property

**Figure 5:**
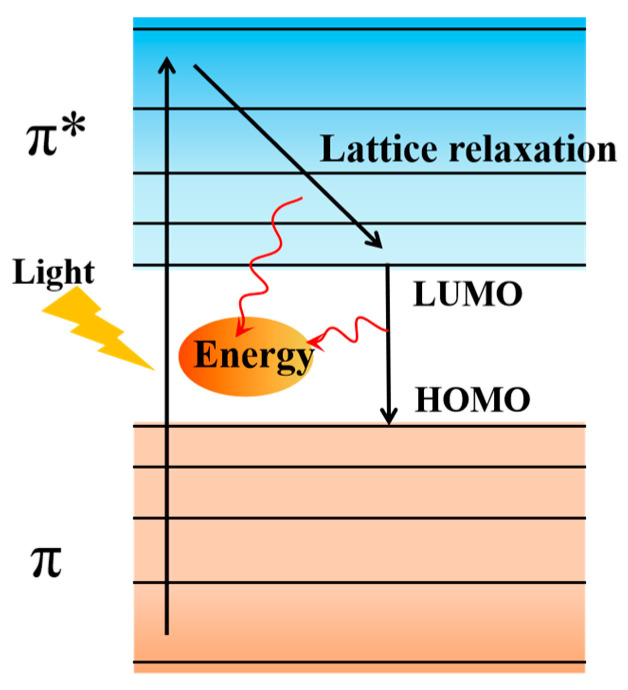
Schematic diagram of the heat production mechanism under PDA light conditions. Reprinted with permission from ref. [79]. Copyright Elsevier, 2021.

The molecular structure of polydopamine is similar to melanin. The PDA structure contains a large number of benzene rings and indole conjugate structures, which have good absorption capacity for visible light, ultraviolet light and near–infrared light, and can effectively convert the absorbed light into heat. Combining this photothermal property of PDA with membrane distillation can effectively solve the drawback of large energy consumption of membrane distillation and expand the application scope of membrane distillation [77,78]. These membrane distillations, which use abundant and renewable solar energy, are called photothermal membrane distillation (PMD) by using photothermal materials to locally heat the feed interface of the membrane rather than heating a large amount of the feed solution [79]. The heat production mechanism of dopamine under light conditions is similar to that of carbon–based materials (Figure 5). Under illumination conditions, electrons can be excited from the ground state with the highest occupied molecular orbital (HOMO) to a higher energy orbital with the lowest unoccupied molecular orbital (LUMO) when the photon energy of the incident light coincides with the electron leap in the molecule. The excited electrons release energy during their re-entry to the ground state through lattice relaxation, resulting in a macroscopic increase in material temperature [80]. The transition of electrons from σ to σ^∗^ orbitals is not possible under the action of light, but because the electron energy within π orbitals is usually weaker than that of σ orbitals, the energy gap between HOMO and LUMO decreases as the number of π bonds increases, and a large number of loose electrons can easily be excited from π to π^∗^ orbitals at a lower energy input [81]. 

**Figure 6:**
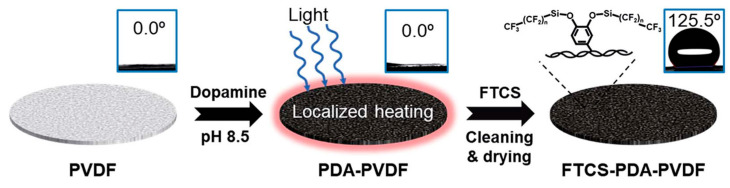
Schematic diagram of the process of preparing a photothermal film with spraying using PDA. Reprinted with permission from ref. [83]. Copyright Royal Society of Chemistry, 2018.

The PMD process avoids the heating of the entire bulk feed water and the process of transporting the feed water from the heating unit to the membrane module, thus saving significant amounts of energy. Direct heating of the membrane surface during membrane distillation essentially alleviates the temperature polarization on the membrane, thus solving the challenge of obtaining clean water using green energy [82]. Wu et al. [83] prepared a simple, stable and efficient solar–driven MD photothermal membrane by polymerizing PDA on a commercial PVDF hydrophilic membrane with spraying (Figure 6), followed by hydrophobic modification of the PDA membrane with 1H,1H,2H,2H–perfluorooctyl trichlorosilane (FTCS). The membrane exhibited an energy efficiency of 45% and a water flux of 0.49 kg/(m^2^·h) at 0.75 kW/m^2^ of solar radiation using a DCMD system. Huang et al. [84] used dopamine to load rGO onto the surface of PTFE hydrophobic membranes and prepared PDA–rGO/PTFE membranes with a 78.6% higher MD overwater flux than the original PTFE membranes under normal solar irradiation, and these experimental results provide new insights into the use of photothermal membrane distillation for seawater desalination.

### 3.4. The Nucleophilic Reaction Properties

**Figure 7:**
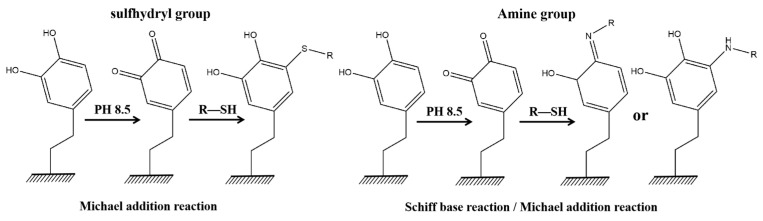
Schematic diagram of the nucleophilic reaction mechanism (Michael addition reaction and Schiff base reaction). Reprinted with permission from ref. [86]. Copyright American Chemica Society, 2013.

Nucleophilic reactions (Michael addition reactions and Schiff base reactions) are important chemical properties of PDA, allowing molecules with terminal primary amine and sulfhydryl functional groups to be grafted onto its surface. Molecules containing sulfhydryl groups can only undergo Michael addition reactions with PDA, whereas molecules containing primary amines can not only react with the Schiff base of the carbon–oxygen double bond of the PDA molecule but can also graft onto the benzene ring structure of the PDA molecule via Michael addition reactions (Figure 7) [85,86].

**Figure 8:**
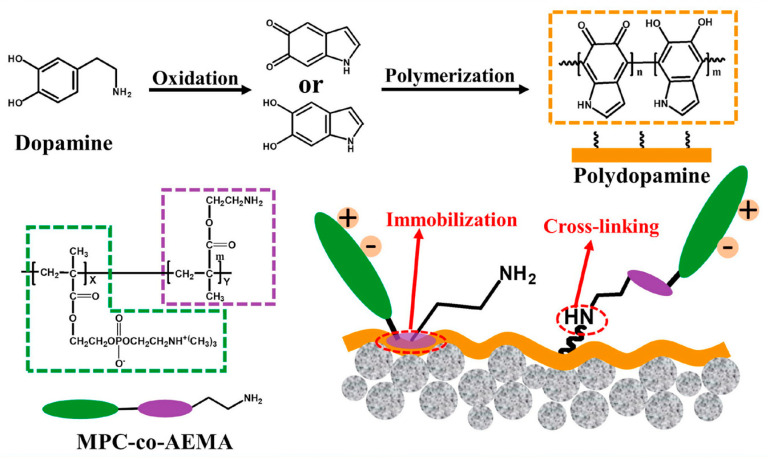
Schematic diagram of the nucleophilic reaction process of PDA with MPC–co–AEMA. Reprinted with permission from ref. [88]. Copyright Elsevier, 2021.

Chen et al. [87] prepared a Janus membrane (PVDF–P–CQD) inspired by nanofiltration (NF) for MD applications by co–depositing a thin film of PDA/PEI on a hydrophobic PVDF membrane and grafting a new zero–dimensional (0D) nanomaterial of sodium functionalized carbon quantum dots (Na^+^–CQDs) with strong hydrophilicity and abundant functional groups on its surface. In DCMD experiments, PVDF–P–CQD membranes showed excellent resistance to wetting and oil contamination, and this study may provide useful insights and strategies for designing a new generation of MD desalination membranes. Zhang et al. [88] prepared PDA/MPC–co–AEMA composite MD membranes with a Janus structure by co–depositing polydopamine/poly MPC–co–2–aminoethyl methacrylate hydrochloride (PDA/MPC–co–AEMA) on the surface of hydrophobic PVDF hollow fibre membranes (HFM) (Figure 8). The functional groups such as amino groups on the surface are covalently bonded to polydopamine. The modified membranes showed good performance against protein contamination and calcium ion fouling using DCMD experiments while improving the organic anti–pollution performance and inorganic salt scale inhibition of the original membranes.

## 4. Mechanism of the Dopamine–Modified Membrane to Improve MD Performance

The membrane distillation process uses a hydrophobic membrane substrate and is driven by the temperature difference across the membrane, with the consequent membrane surface contamination and temperature polarization becoming the main factors affecting the membrane distillation performance. The deposition of PDA on the membrane surface can alleviate the membrane contamination and temperature polarization phenomenon during membrane distillation operation. Firstly, PDA contains many special functional groups, which makes the membrane surface with universal adhesion and rich post–functionalization ability, and improves the MD performance by loading other functional materials with excellent properties. Then, the loading of PDA introduces a large number of amino and hydroxyl groups, which improves the membrane usability by constructing a hydrophilic anti–pollution surface. Finally, due to the photothermal properties of PDA, the photon energy absorbed from within the solar spectrum is converted into heat on the membrane surface, thus alleviating the temperature polarization phenomenon on the membrane surface and improving the MD performance.

### 4.1. Adhesion Enhances the Performance of MD Membranes

**Figure 9:**
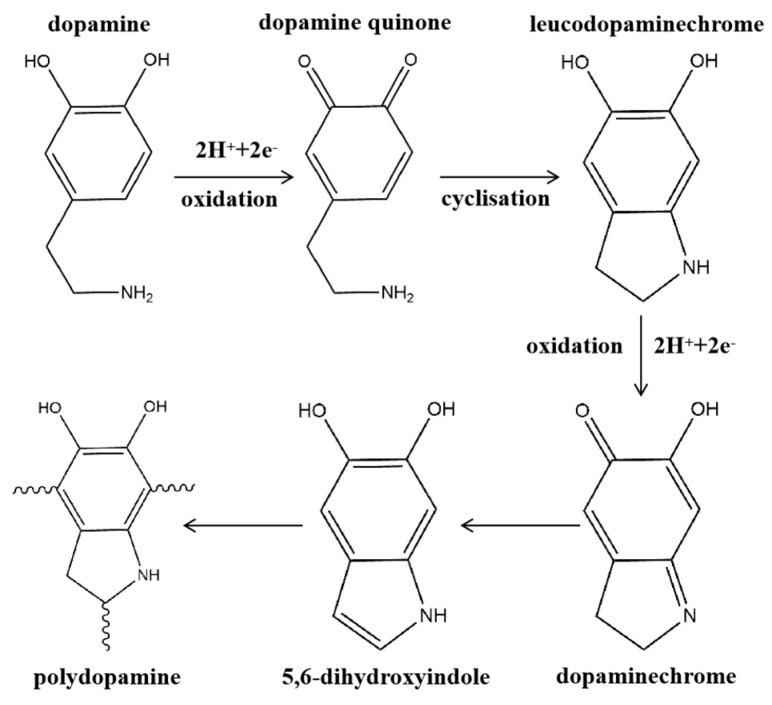
Schematic diagram of the mechanism of oxidative polymerization of dopamine. Reprinted with permission from ref. [91] copyright John Wiley and Sons, 2012.

At present, due to the complex reaction process of dopamine polymerization and deposition on membrane surfaces, there is still no consensus on the reaction mechanism on the membrane surface. It is generally accepted that the deposition process of dopamine on the membrane surface can be divided into two processes: oxidative polymerization, in which dopamine gradually forms a cross–linked network, and deposition, in which PDA interacts with the substrate [89,90]. Despite that researchers are not definitive about the mechanism of dopamine oxidative polymerization, it is generally believed that the oxidative auto–polymerization process of dopamine is as shown in Figure 9. Dopamine is first oxidized to a quinone structure, and then the primary amine undergoes intramolecular cyclization with a nucleophilic reaction, followed by an intramolecular rearrangement reaction to produce 5,6–dihydroxy indole, and finally auto–polymerization [91,92]. During the oxidative polymerization process, dopamine needs to be exposed to an oxidizing environment with alkaline or added oxidants, and after oxidative polymerization, it is further assembled using non–covalent forces into a polymeric aggregate called PDA. This polydopamine does Brownian motion in the solution, and when the motion reaches the membrane surface, the special functional groups in PDA such as catechol, o–dibenzoquinone, amino and carboxyl groups interact with the membrane surface by force, which makes PDA deposited on the membrane surface. The complex molecular structure and rich functional groups of PDA endow the PDA–modified membrane with pervasive adhesion and rich post–functionalization capabilities, which enable the PDA–modified membrane to further load functional materials with excellent performance on the membrane surface and enhance the performance of membranes for membrane distillation [93,94].

### 4.2. Hydrophilicity Enhances Contamination Resistance of MD Membranes

**Figure 10:**
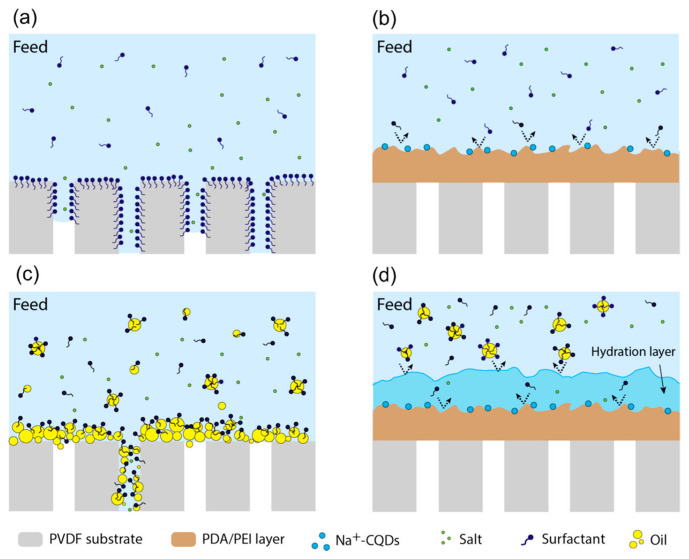
Schematic diagram of dopamine–modified membranes to resist fouling in membrane distillation: (**a**) PVDF membranes with surfactant–based contaminants; (**b**) modified membranes with surfactant–based contaminants; (**c**) PVDF membranes with oil–based contaminants; (**d**) modified membranes with oil–based contaminants. Reprinted with permission from ref. [87]. Copyright American Chemica Society, 2021.

In the process of membrane distillation, when treating water polluted with amphiphilic substances containing surfactants (such as printing and dyeing wastewater, laundry wastewater, etc.), hydrophobic MD membranes such as unmodified PVDF and PTFE are highly susceptible to adhesion and wetting of surfactants such as sodium dodecyl sulphate (SDS) [95,96]. There are two main reasons for the wetting; on the one hand, the SDS accumulates at the water–air interface on the surface of the hydrophobic membrane, reducing the surface tension and contact angle of the incoming liquid, making the incoming liquid pressure LEP value of the membrane lower than the water pressure difference across the membrane, thus allowing the liquid to invade the membrane pores. On the other hand, as surfactants are amphiphilic chemicals containing a hydrophilic head and a hydrophobic tail, SDS molecules adsorb to the membrane pore surface through attractive hydrophobic–hydrophobic interactions due to the presence of the hydrophobic tail (Figure 10a). This results in the exposure of their hydrophilic heads to water and makes the otherwise hydrophobic membrane pore surface hydrophilic, thus allowing the influx of the feed solution into the wetted membrane pores and causing the MD process to fail [97,98,99,100]. The deposition of a PDA–modified layer on the hydrophobic membrane surface can largely improve the anti–wetting performance of the membrane in MD, which is mainly due to the size rejection effect of the PDA layer on the surfactant molecules. The presence of the modified layer reduces the pore size of the original membrane, and the PDA layer acts as a sieve during the MD operation, preventing the contact of surfactant molecules with the hydrophobic substrate (as shown in Figure 10b), thus effectively alleviating the membrane wetting.

**Figure 11:**
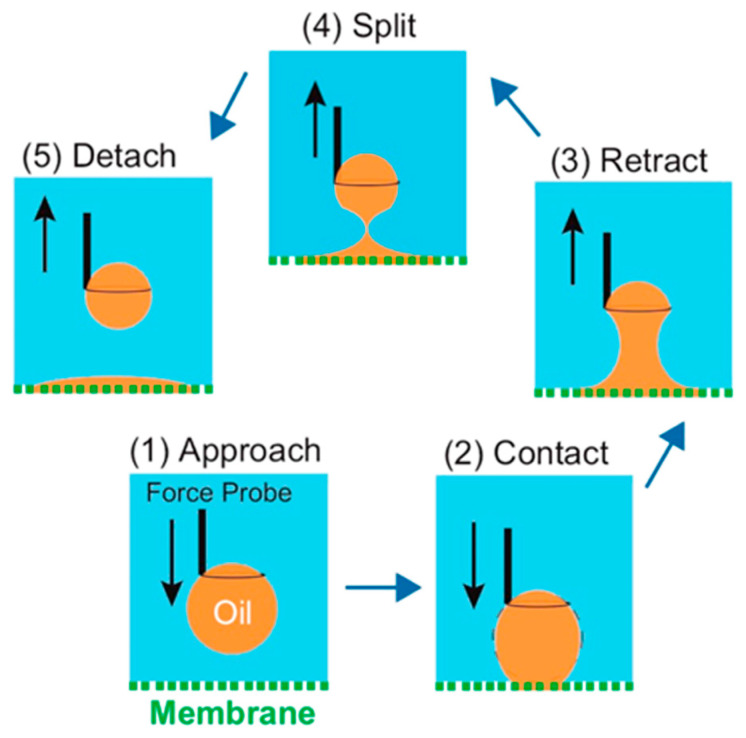
The experiment of underwater adhesion measurement between membrane and oil droplets. Reprinted with permission from ref. [101]. Copyright American Chemica Society, 2016.

For oily water bodies, the strong hydrophobic interaction between the oil contaminants and the hydrophobic membrane still leads to oil adhering to the membrane surface and entering the membrane pores, causing MD membrane wetting (as shown in Figure 10c). Wang et al. [101] elucidated the tendency of conventional MD membranes to contaminate in oily feed liquids from a mechanical perspective by measuring the underwater adhesion between hydrophobic membrane surfaces and oil droplets. This measurement is divided into five processes (as in Figure 11), and by default the adhesion force is zero in process (1). In process (2), the adhesion force increases abruptly due to the strong hydrophobic–hydrophobic interaction between the oil droplets and the membrane surface. In process (3), the adhesion force gradually increases as the oil droplets continue to be stretched. A sharp decrease in the adhesion force can be detected when the oil droplets are finally separated from the membrane surface in process (4). For process (5), the recorded adhesion force differs from that of process (1), which can be attributed to the fact that the final remaining oil droplets become smaller and their buoyancy is lower than that of the original larger droplets, indicating the tendency of oil droplets to contaminate the membrane surface. The construction of a PDA layer on the surface of hydrophobic membranes will greatly improve the anti–pollution performance of membranes against oily water, mainly because dopamine contains many hydroxyl groups, amino groups and other hydrophilic groups, which will form hydrogen bonds with water molecules when in contact with them, thus forming a hydrated layer on the membrane surface (as shown in Figure 10d) [102]. The presence of the hydration layer hinders the attachment of oil droplets to the hydrophobic membrane surface, allowing the PDA–modified membrane to have a low tendency to contaminate in MD operations.

### 4.3. Photothermal Properties to Relieve Temperature Polarization in MD Membranes

**Figure 12:**
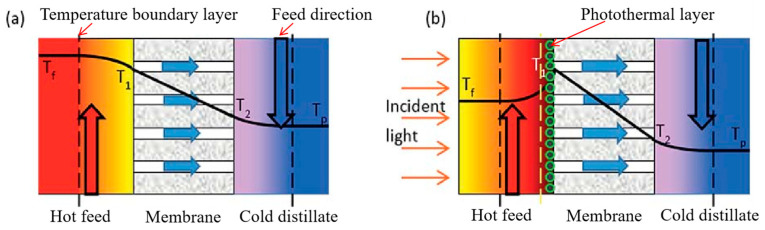
Schematic diagram of temperature difference polarization between conventional membrane distillation and photothermal membrane distillation: (**a**) conventional membrane distillation; (**b**) photothermal membrane distillation. Reprinted with permission from ref. [104]. Copyright Royal Society of Chemistry, 2017.

In conventional membrane distillation, the evaporation of water and the temperature polarization caused by the thermal conductivity of the membrane significantly limit the thermal efficiency of MD [103]. Due to temperature polarization, the membrane surface temperature on the feed side (*T*_1_) may be significantly lower than the temperature of the feed side feed (*T*_f_*)*, while the membrane surface temperature on the condensing side (*T*_2_) may be significantly higher than the temperature of the cold side condensate (*T*_p_) (Figure 12a). This temperature change significantly reduces the transmembrane vapor pressure difference, and this change is known to reduce the MD flux with Equation (1). And in some cases, the temperature polarization coefficient (α_TP_) in Equation (2) may be as low as 0.3, which indicates that temperature polarization leads to a 70% reduction in the effective driving force of membrane distillation [104].

(1) $$J=k(P_{1}-P_{2})$$

where *J* is the membrane distillation water flux (m·s^−1^); *k* is the membrane water vapor transmission rate (m·s^−1^·pa^−1^); *P*_1_ and *P*_2_ are, respectively, for the feed measurement and condensation side of the water–gas interface vapor pressure difference (pa).

(2) $$\alpha_{TP}=\frac{T_{1}-T_{2}}{T_{f}-T_{p}}$$

In contrast, combining dopamine with a membrane surface meanwhile uses the photothermal properties of dopamine, which can absorb photonic energy from most of the light within the solar spectrum as well as convert it locally and efficiently into thermal energy [105]. Therefore, the PDA membrane surface temperature (*T*_1_) on the feed side may be significantly higher than that of the feed side feed (*T*_f_ (Figure 12b), which increases the transmembrane vapor pressure difference and temperature polarization coefficient driving MD, thus alleviating the temperature polarization phenomenon and enhancing the MD flux. In addition to this, Wu et al. [83] prepared a photothermal MD film using PDA with a photoconversion efficiency of 45% when treating salt water at a light intensity of 0.75 kW/m^2^, which is higher than the photoconversion efficiency of photothermal films prepared using silver nanoparticles (36.9%) [106], nitrocellulose (31.8%) [107] and carbon black (21.5%) [108]. The main reason for this is that PDA contains a large number of benzene rings and indole–like conjugated structure, making it a good absorption of visible light, ultraviolet light and near–infrared light with a wider range of light absorption than other materials. Secondly, PDA can convert 99% of the absorbed photon energy into thermal energy within 50 ps, which has excellent light conversion performance.

## 5. Conclusions and Outlook

Dopamine exhibits great potential for membrane distillation. The adhesive and hydrophilic properties of dopamine simplify the hydrophilic modification of the membrane, greatly improving the membrane’s resistance to contamination during the MD process. By utilizing the photothermal properties of PDA, the temperature polarization can be alleviated, leading to improved processing efficiency and reduced operational costs in MD. In addition, the post–functionalization capability of PDA can be combined with the outstanding properties of other materials, which further broadens the application range of MD films. Yet, there are many issues concerning dopamine in membrane distillation applications that require further research.

1. Stability of polydopamine coatings. In membrane distillation, hydrophobic organic polymer films are generally used, and dopamine is deposited on their surface with polymerization through non–covalent bonds. During the operation of membrane distillation, the stability of the coating reflects the performance of the membrane distillation. The modified layer on the membrane surface is tested with contaminant adhesion, water washout and temperature changes, etc. How to effectively improve the long–term stability of the coating is one of the future research directions.
2. Adhesion mechanisms of polydopamine coatings. Currently, dopamine enhances the performance of membrane distillation in the treatment of various wastewater processes by co–depositing with other modifiers on the membrane surface through its universal bonding property and post–functionalization ability, but its self–polymerization mechanism and co–deposition mechanism with other modifiers are still not fully explained. The lack of final conclusions regarding the polymerization process, the exact structure and the composition of dopamine still hinder further improvement of the performance of membranes for membrane distillation.
3. Modification methods for polydopamine coatings still have limitations. Traditional methods of dopamine deposition require long modification times; uneven modification of the membrane surface and physical deposition can result in a waste of dopamine drugs. Although the researchers accelerated the deposition of dopamine and improved the uniformity of the film surface by using different oxidants, UV light and ultrasound radiation, these methods only accelerated the Brownian motion of dopamine aggregates and still did not control the direction of dopamine deposition. These issues limit the widespread use of dopamine in membrane distillation. How to solve the problem of directional deposition of PDA and how to apply it in membrane distillation on a large scale is still a problem that needs to be explored.

## Data Availability

The original contributions presented in the study are included in the article, further inquiries can be directed to the corresponding author.
